# Kumatakenin inhibited iron-ferroptosis in epithelial cells from colitis mice by regulating the Eno3-IRP1-axis

**DOI:** 10.3389/fphar.2023.1127931

**Published:** 2023-03-17

**Authors:** Arenbaoligao, Xinrui Guo, Jiahao Xiong, Shuangshuang Zhang, Yuewen Yang, Dapeng Chen, Yu Xie

**Affiliations:** ^1^ Department of Pharmacy, The Second Affiliated Hospital of Dalian Medical University, Dalian, China; ^2^ Comparative Medicine Department of Researching and Teaching, Dalian Medical University, Dalian, China; ^3^ Shandong First Medical University, Jinan, China

**Keywords:** kumatakenin, colitis, ferroptosis, iron level, ENO3

## Abstract

Inhibition of epithelial ferroptosis in colonic tissues relieved clinical symptoms and improved endoscopic presentations in inflammatory bowel disease (IBD). Kumatakenin, the main ingredient of traditional Chinese medicinal cloves and *Alpinia purpurata*, is reported to possess therapeutic benefits. However, whether kumatakenin could inhibit ferroptosis and further alleviate colitis remains unclear. Here, we measured the effects of kumatakenin on ferroptosis of colonic epithelial cells from colitis mice. The colitis model was induced in mice by oral intake of 2.5% dextran sulfate sodium in drinking water. RNA sequencing was performed to investigate the mechanism underlying kumatakenin-mediated effects on colitis. The results showed that different doses of kumatakenin significantly alleviated symptoms and suppressed intestinal inflammation in the colitis mouse model. Kumatakenin supplementation decreased cellular iron levels and suppressed ferroptosis in epithelial cells from colitis mice. RNA sequencing, qPCR, and pharmacological inhibition assays showed that kumatakenin reduced cellular iron levels and suppressed ferroptosis in epithelial cells from colitis mice at least partially by upregulating expression of enolase (Eno-3). Furthermore, kumatakenin decreased iron levels in epithelial cells by modulating the Eno3-iron regulatory protein (IRP1) axis. Molecular docking results revealed that kumatakenin could bind Eno3 *via* hydrogen bonding with the amino acid residues Thr208, Val206, and Pro203. This work will provide a scientific basis for the clinical use of kumatakenin in the treatment of colitis.

## Introduction

Ulcerative colitis (UC) and Crohn’s disease (CD), the two clinical classifications of inflammatory bowel disease (IBD), are complex chronic inflammatory conditions characterized by severe diarrhea, abdominal pain, fatigue, and weight loss (Fitzpatrick et al., 2022). IBD has emerged as a public health challenge worldwide during the past decades (Kaplan and Windsor, 2021). More than 2 million and 1.5 million people are suffering from IBD in Europe and North America, respectively (Zhao et al., 2021).

Pathologies of UC mainly involve the mucosa of the large intestine (Kushkevych et al., 2021); however, its precise etiology remains largely unclear. Ferroptosis, a novel non-apoptosis programmed cell death, is accompanied by the accumulation of iron and lipid peroxidation during the cell death process (Jiang et al., 2021; Liang et al., 2022). Colonic epithelial cells from human UC and dextran sulfate sodium (DSS)-induced murine colitis exhibited increased lipid peroxidation and ferroptosis (Mayr et al., 2020; Li et al., 2022). Iron chelator administration significantly reduced lipid peroxidation in colonic tissues of UC patients, ameliorated clinical symptoms, and improved endoscopic presentations, suggesting the possibility of developing therapeutic strategies for UC (Ellermann et al., 2020; Di Paola et al., 2022).

Ferroptosis is iron-dependent; accumulation of cellular iron leads to increased oxidation of various macromolecules and the onset of ferroptosis (Huang et al., 2022; Stockwell, 2022). Iron transport, storage, and regulatory proteins regulate cellular iron homeostasis precisely (Rennekamp, 2017; Gao et al., 2022). Iron regulatory protein 1 (IRP1) is mammalian protein reported to regulate iron metabolism-related gene expressions to optimize cellular iron availability at the post-transcriptional level (Maio et al., 2021).

Enolase (ENO), a member of the RNA degradosome in *Escherichia coli*, promotes degradation of RNA (Carpousis, 2007). In particular, ENO promotes mRNA degradation of IRP1, leading to reduction of cellular iron levels in cancer cells, and finally protects cancer cells from ferroptotic cell death (Zhang et al., 2022).

Kumatakenin, the main components of traditional Chinese medicinal cloves and *Alpinia purpurata*, possesses several therapeutic properties. Kumatakenin exhibits anti-cancer activities by inducing cancer cell death and suppressing the polarization of tumor-associated macrophages (Woo et al., 2017). Kumatakenin promotes antibiotic-exerted effects used to treat pathogenic bacteria (Zhang et al., 2019). Kumatakenin also inhibits SARS-CoV-2 replication in Vero E6 and Calu-3 cells (Leal et al., 2021). Finally, kumatakenin has been shown to suppress cytokine production (Villaflores et al., 2010). However, the influence of kumatakenin on IBD remains unclear.

Here, we measured the effects of kumatakenin on DSS-induced colitis and determined whether kumatakenin inhibited ferroptosis in colonic epithelial cells by reducing iron levels.

## Material and methods

### Animals

C57BL/6 mice (male, 28–35 g) were purchased from Cyagen Biosciences Inc. (Suzhou, China). All mice were maintained under specific pathogen-free conditions at tge Experimental Animal Center, Dalian Medical University (Certificate of Conformity SYXK Liao 2013–0006). The study was approved by the Dalian Medical University Animal Care and Ethics Committee (No: AEE20046). The animals had *ad libitum* access to food and water for 2 weeks prior to experimentation in laboratory conditions of 23°C, 12 h/12 h light/dark, and 50% humidity. No animals died before the study was started.

### Reagents

Kumatakenin (PubChem CID: 5318869) of ≥95% purity and sulfasalazine (SASP, PubChem CID: 5359476) were obtained from Sigma-Aldrich (St. Louis, MO, United States of America). Anti-enolase (Eno3, ab232759), anti-iron regulatory protein (IRP1, ab183721), anti-4-HNE (ab48506), and anti-GAPDH (ab9485) antibodies were obtained from Abcam (Hong Kong, China). Other reagents were purchased from Sigma-Aldrich.

### Experimental design

Acute colitis was induced in C57BL/6 mice with 2.5% dextran sulfate sodium (DSS) dissolved in sterile distilled water for 9 days. Mice were randomly assigned to eight groups of 12 animals each. Control mice in group I were given sterile distilled water. Groups II, III, IV, and V were treated with DSS for 9 days to induce colitis. Group II was a DSS colitis model control. Mice in groups III, IV, and V drank sterile distilled water containing DSS and were treated by intragastric administration of 150 mg/kg SASP, low-dose 25 mg/kg kumatakenin, or high dose 100 mg/kg kumatakenin (dissolved in sterile distilled water) for 9 consecutive days, respectively.

Groups Ⅵ, Ⅶ, and Ⅷ were administered Eno3 inhibitor ENOblock (1 mg/kg) by intraperitoneal injection once a day for 3 days to inhibit Eno3 expression *in vivo*. Group Ⅵ served as the Eno3 depletion control. Following pretreatment with ENOblock, Group Ⅶ was given DSS for 9 consecutive days. Mice in group Ⅷ received water containing DSS and were intragastrically administered 100 mg/kg kumatakenin (dissolved in sterile distilled water) for 9 consecutive days in the presence of Eno3 depletion. SASP is an anti-inflammatory drug used to treat IBD and was a positive control for the effects of kumatakenin on colitis.

### Assessment of colitis symptoms

Colon histology, morphology, and the severity of inflammation were assessed by colonoscopy, HE staining, and colon length sections. Disease activity index, rectal bleeding, diarrhea, and daily food intake/body weight were recorded.

Disease activity index (DAI) scores have historically correlated well with pathological findings in a DSS-induced model of IBD (Ghia et al., 2008). The DAI is the combined score of weight loss, stool consistency, and bleeding. Scores were defined as follows: body weight loss, 0, no loss; 1, 5%–10%; 2, 10%–15%; 3, 15%–20%; 4, 20%; stool consistency, 0, normal; 2, loose stool; 4, diarrhea; bleeding, 0, no blood; 2, presence of bleeding; and 4, gross bleeding.

An enzyme-linked immunosorbent assay (ELISA) was performed to measure the production of colonic mediators and cytokines. Harvested colon tissue specimens were cut into 5 mm pieces, processed, and mounted on slides for immunohistochemical analysis.

### Cell viability

The viability of MODE-K cells was determined by measuring cellular metabolism using 96-well plates. A total of 1 × 10^6^ MODE-K cells was seeded into 96-well plates in 10% FBS medium per well and incubated overnight (37°C, 5% CO_2_). Medium was replaced the next day with DMEM containing different concentrations of kumatakenin (10 or 80 μM) in the presence of different doses of erastin (0.1, 0.2, 0.5, 1, or 10 μM). After treatment for 48 h, cells were incubated with CCK8 solution for 4 h. Finally, absorbance was measured at 450 nm using a microplate reader.

### Transmission electron microscopy

Colonic tissue from mice in different groups was fixed with glutaraldehyde (2.5%), and then post-fixed in osmium tetroxide (2%) with potassium ferrocyanide (1.6%) in sodium cacodylaten (0.1 mol/L). Colonic tissue samples were then cut to 5 mm^3^ sections, stained with 2% uranyl acetate, dehydrated in increasing concentrations of acetone (50, 70, 80, and 90%) twice, and embedded in eponate. Sections were further cut into 80 nm sections, and stained with hematoxylin and eosin (HE), uranyl acetate (2%), and lead citrate. A Hitachi H7600 transmission electron microscope was used to capture images in the microscope core.

### Flow cytometry

MODE-K cells in different groups were incubated with BODIPY 581/591 C11 (5 μM) at 37 °C for 30 min. Digested solutions were strained and pelleted to isolate cells. Cells were washed, resuspended in phosphate-buffered saline (PBS), transferred through a cell strainer for flow cytometry (The BD Accuri™ C6 Plus).

### RNA sequencing (RNA_seq)

Total RNA from colonic tissues from the DSS control and DSS plus kumatakenin group were extracted using TRIzol reagent according to the manufacturer’s instructions. Using the protocol for the mRNA-Seq sample preparation kit, a final cDNA library was established. Mean insert size for the paired-end libraries was 150 bp. The RNA_seq data presented in this study have been deposited in Sequence Read Archive database with the accession number PRJNA913780 (https://dataview.ncbi.nlm.nih.gov/object/PRJNA913780?reviewer=idsh27k1p7lf43eqduqn2b0hsg).

### Iron determination

To assess iron at the tissue level, colonic tissue (10 mg) from mice in different groups was isolated and washed with cold PBS. Homogenization was performed using a homogenizer in 4–10 volumes of Iron Assay Buffer using a Dounce on ice, with 10–15 passes. Tissue homogenates were centrifuged at 16,000 × *g* for 10 min to remove insoluble materials, and the supernatant was prepared for iron determination following the iron assay (Abcam, Cambridge, UK; ab83366).

To assess iron at the cellular level, 10^6^ cells were washed with PBS and lysed in 50 μL of lysis buffer containing 1% Triton X-100. Cell lysates were centrifuged at 15,000 rpm for 10 min, and the supernatants were used to measure total iron labile (Fe^2+^ and Fe^3+^) according to the manufacturer’s instructions. The Quantichrom iron assay (BioAssay Systems, Hayward, CA, United States of America; DIFE-250) was used for studying cellular iron levels at 590 nm.

### The mRNA stability assay

MODE-K cells incubated with complete DMEM were treated with actinomycin D for 0, 3, or 6 h. Total RNA was collected with TRIzol reagent, and mRNA levels were analyzed by qPCR. qPCR reactions were performed in triplicate using Monet Mix and specific assay kits. Amplification was performed for 45 cycles with enzyme activation at 95°C for 30 min followed by denaturation at 60°C.

### Small interfering RNA (siRNA) silencing of Eno3 and IRP1

A total of 30 nmol/L of Eno3 and IRP1 siRNAs (GENERAL BIOL, Chuzhou, Anhui) was introduced into MODE-K cells by transient transfection with Lipo2000 according to the manufacturer’s instructions. Cells were collected 48 h after transfection and levels of Eno3 and IRP1 expression were measured.

### Molecular docking

The 3D structure of Eno3 (PDB ID: 2XSX) was downloaded from the Protein Data Bank. As previously described (He et al., 2020), the 3D structure of kumatakenin was initially constructed using Chem3D software (Cambridge Soft, Waltham, MA, United States of America), and optimized with Discovery Studio software (BIOVIA Inc., San Diego, CA, United States of America) at CHARMmforce field. Finally, the CDOCKER protocol of Discovery Studio was used to analyze the interaction of kumatakenin and Eno3.

### Statistical analysis

Animal experiments, *in vitro* experiments, and data analysis were conducted according to a single-blind study design. One-way analysis of variance was used for multiple group comparisons; multiple comparison between groups was performed using the S-N-K method. Two-tailed unpaired Student’s *t*-tests were used to evaluate between two group comparisons. Data are expressed as means ± standard deviation (SD). The data were normally distributed and the groups had equal variances. All experiments were repeated at least three times. *p*-values less than 0.05 were considered statistically significant.

## Results

### Dose selection and toxicity of kumatakenin in normal cells and mice

Cytotoxicity of kumatakenin in MODE-K cells was measured to verify the effective concentration of kumatakenin *in vitro*. Our results showed that kumatakenin exerted no significant cytotoxicity on MODE-K cells at the dose range of 5–80 μΜ (Supplementary Figure S1A). At 10–120 mg/kg, gastric administration of kumatakenin induced no significant alteration of body weight (Supplementary Figure S1B), disease activity index (Supplementary Figure S1C), colonic proinflammatory cytokine TNF-α production (Supplementary Figure S1D), serum FD-4 content (Supplementary Figure S1E), colonic iron level (Supplementary Figure S1F) or colonic expression of Eno3 (Supplementary Figure S1G) in normal mice.

### Kumatakenin alleviated DSS-induced mice colitis symptoms

**FIGURE 1:**
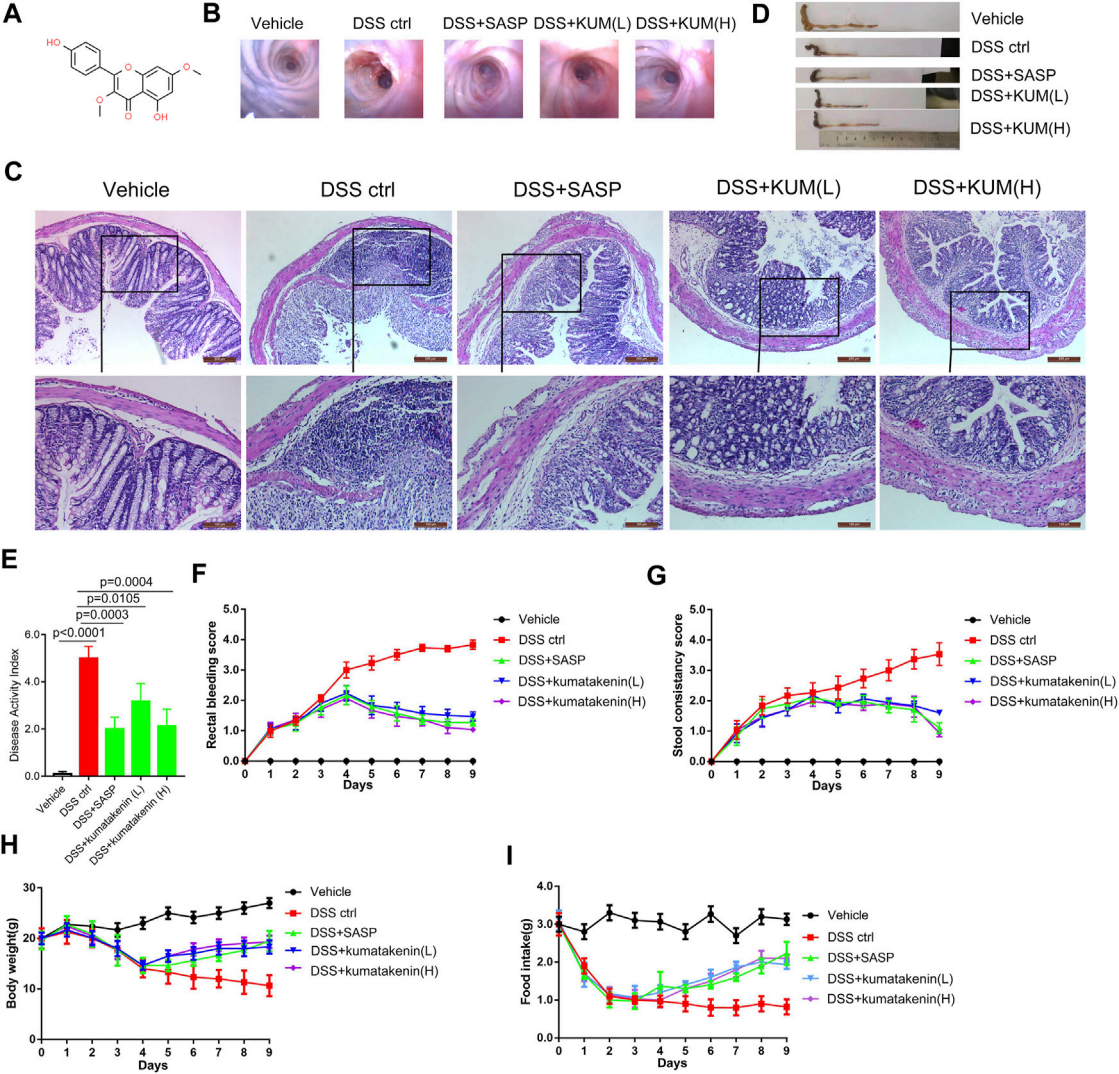
Effects of kumatakenin treatment on DSS-induced colitis in mice. **(A)** Chemical formula of kumatakenin. Mice were administered DSS to induce colitis. **(B)** Colonoscopy and **(C)** hematoxylin and eosin (HE) staining of mice colonic tissue was performed to measure the effects of kumatakenin on vascular patterns, atrophy of intestinal villi, and infiltration of inflammatory cells (above: scale bar = 200 μm; below: scale bar = 100 μm). Effects of kumatakenin on **(D)** colon length, **(E)** disease activity index, **(F)** rectal bleeding score, **(G)** stool consistency score, **(H)** body weight, and **(I)** food intake in mice with colitis. Data are expressed as the mean ± standard deviation (SD). SASP, sulfasalazine; kumatakenin (L), 25 mg/kg kumatakenin; kumatakenin **(H)**, 100 mg/kg kumatakenin.

**FIGURE 2:**
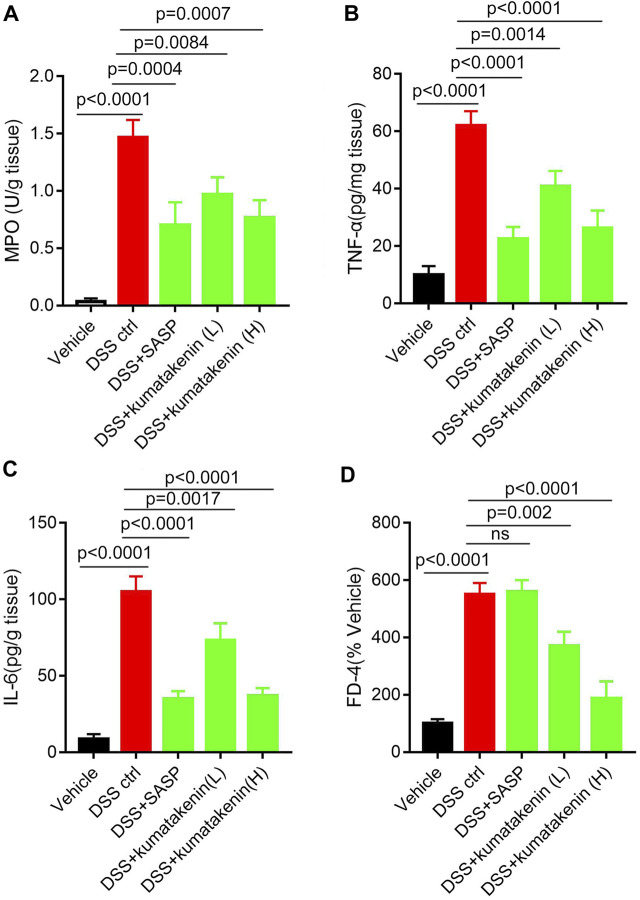
Effects of kumatakenin on colonic inflammation and epithelial barrier function in colitis mice. Mice with colitis were gavaged with kumatakenin (25 mg/kg/day; 100 mg/kg/day) for 9 days and **(A)** colonic MPO level, **(B,C)** pro-inflammatory cytokines (TNF-α and IL-6) and **(D)** serum fluorescein isothiocyanate-dextran (FD-4) levels were measured. SASP, sulfasalazine; kumatakenin (L), 25 mg/kg kumatakenin; kumatakenin (H), 100 mg/kg kumatakenin.

As shown in Figures 1B, C, colonoscopy and HE staining showed mucosal bleeding, vascular patterns, infiltration of inflammatory cells, and atrophy of intestinal villi in colonic tissues from DSS-treated mice. Excessive intestinal inflammation was also observed after DSS treatment, which was characterized by short colon length (Figure 1C), DAI (Figure 2E), rectal bleeding (Figure 2F), stool consistency score (Figure 2G), and developed body weight/food intake loss (Figures 2H, I). These data confirmed successful establishment of a colitis model.

Oral administration of kumatakenin and SASP alleviated mice colitis symptoms, including restoration of mucosal bleeding, vascular patterns, infiltration of inflammatory cells, and atrophy of intestinal villi. Increased colon length, decreased DAI score, alleviation of diarrhea and rectal bleeding, and increased body weight/food intake was observed in kumatakenin-treated colitis mice. Overall, these data showed that kumatakenin significantly ameliorated colitis in a DSS-induced model.

### Kumatakenin decreased cytokine production and restored epithelial barrier loss in colitis mice

Intestinal inflammation is related to increased neutrophil infiltration and accumulation of proinflammatory cytokines and mediators (Liu et al., 2022). Figure 2 shows that MPO activity (Figure 2A), pro-inflammation cytokine TNF-α/IL-6 (Figures 2B, C) production in colonic tissues, and FD-4 levels (Figure 2D) in serum samples from colitis mice were significantly higher than those from normal mice; these effects could be reversed by kumatakenin or SASP. These data suggested that kumatakenin reduced colonic inflammation and restored barrier function in DSS-treated mice.

### Kumatakenin suppressed ferroptosis in colonic epithelial cells from colitis mice

Enhanced ferroptosis in colonic epithelial cells was observed in chemically induced colitis, and inhibition of ferroptosis has been shown to be beneficial for the treatment of IBD (Mayr et al., 2020). Thus, mitochondrial morphology, colonic iron level, and lipid peroxidation products malonaldehyde and 4-hydroxynonenal (HNE) contents were measured in colonic epithelial cells from colitis.

**FIGURE 3:**
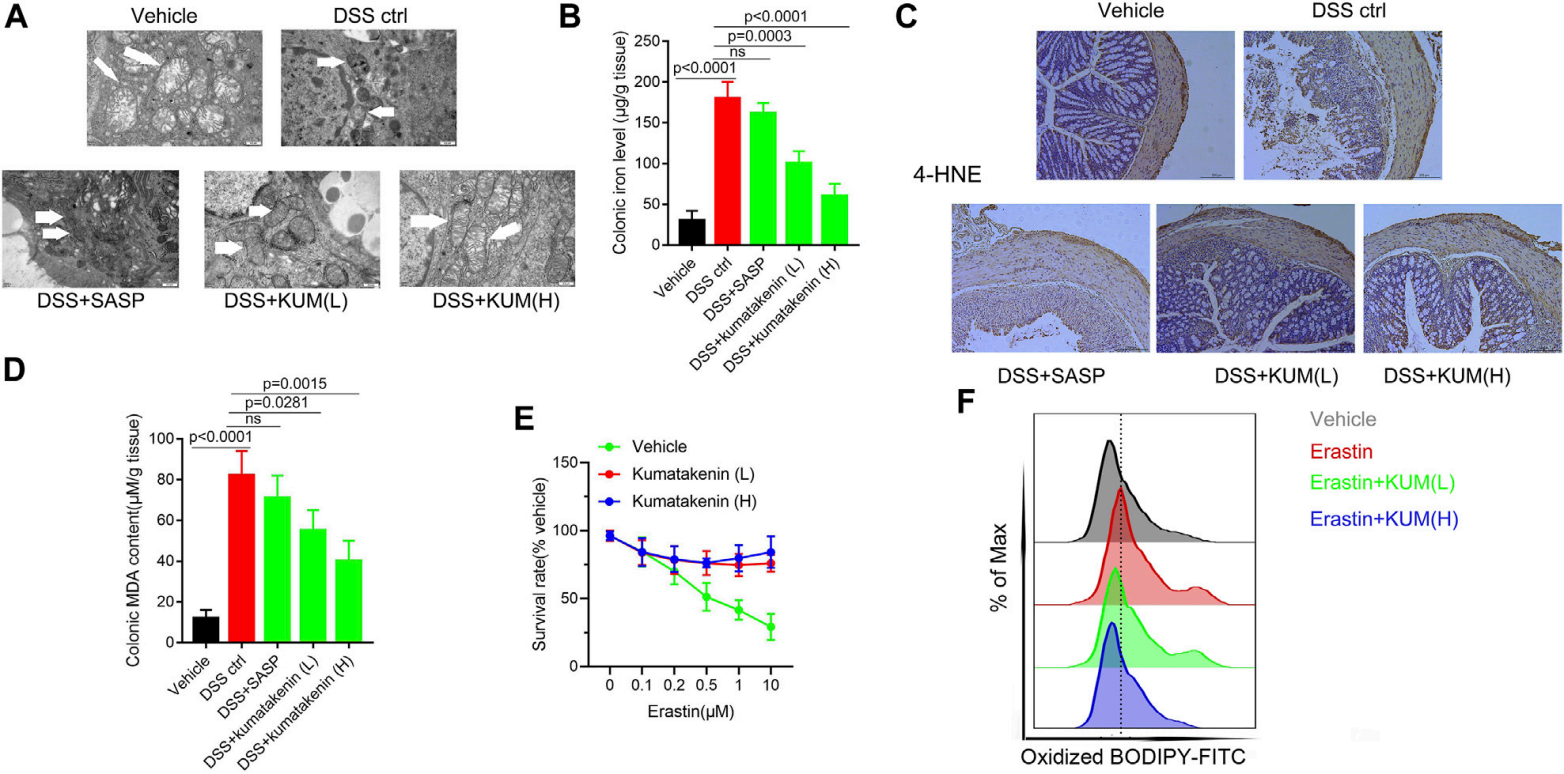
Kumatakenin inhibited ferroptosis in colonic epithelial cells from colitis mice. **(A)** Mitochondrial morphology in colonic epithelial cells from colitis mice was determined using transmission electron microscopy (scale bar = 0.5 μm). **(B)** Colonic iron and **(C,D)** 4-HNE and MDA expression were measured to reflect the lipid peroxidation rate (scale bar = 200 μm). **(E)** Cell survival rate and **(F)** lipid ROS levels of MODE-K cells after ferroptosis inducer erastin treatment were also measured.

Transmission electron microscopy (TEM, Figure 3A) showed the presence of pyknosis of mitochondria in colonic epithelial cells from colitis mice. ELISA (Figure 3B) showed increased colonic iron levels in mice after DSS treatment. In addition, IHC and ELISA showed that increased expression of 4-HNE and higher MDA content were observed in DSS-induced colitis mice (Figures 3C, D). These results suggested that increased ferroptosis was determined in colonic epithelial cells in colitis mice. Kumatakenin administration exerted significant anti-ferroptosis effects in epithelial cells from colitis mice, including restoration of mitochondrial morphology, reduced iron level, and decreased expression of MDA and 4-HNE. The effects of kumatakenin on ferroptosis were also measured *in vitro*. CCK-8 results showed that erastin induced significant cell death; this effect was reversed by kumatakenin (Figure 3E). Flow cytometry results showed increased lipid peroxidation level in the erastin-treated group, which could also be reversed by kumatakenin (Figure 3F). Taken together, kumatakenin treatment exerted significant anti-ferroptosis effects in epithelial cells.

### Kumatakenin decreased epithelial ferroptosis in colitis mice by upregulating Eno3 expression

**FIGURE 4:**
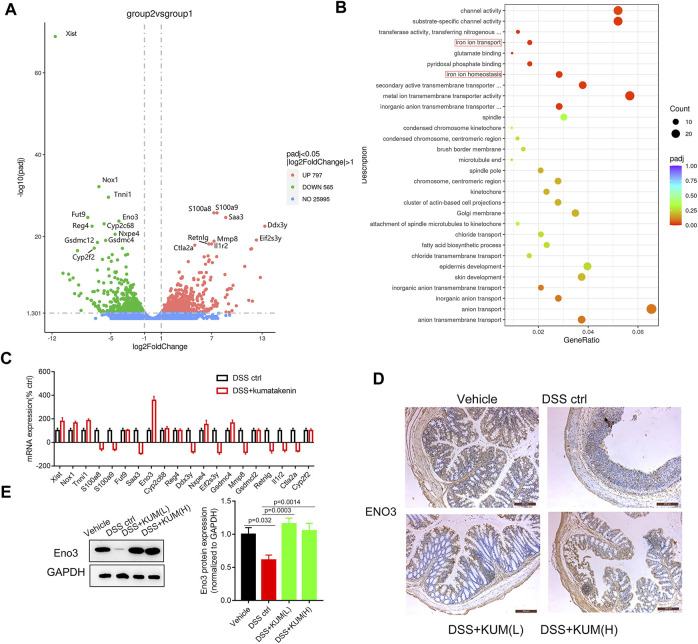
Kumatakenin suppressed ferroptosis in epithelial cells in an Eno-3 dependent manner. **(A)**Volcanic map of differentially expressed genes (DEGs) in colonic tissues from colitis mice following treatment with kumatakenin. **(B)** The most significant enrichment pathways of DEGs as determined by GO analysis. **(C)** qPCR verification of the top 20 most DEGs between the DSS control and DSS + kumatakenin groups. **(D,E)** Effects of kumatakenin on Eno3 protein expression in colitis mice (scale bar = 200 μm).

To measure the underlying mechanism of kumatakenin-exerted suppression of ferroptosis, RNA-seq was performed. Data showed that 797 genes were upregulated and 565 genes were downregulated significantly (P_adj_ < 0.05, |log_2_foldchange|>1) in the kumatakenin-treated group compared with the DSS control group. The top 20 most differentially expressed genes between the DSS control and DSS + kumatakenin groups were chosen for further investigation (Figure 4A). The most enriched pathways of DEGs, including iron ion transport (GO: 0006826) and iron ion homeostasis (GO:0055072) pathways, as determined by Gene Ontology (GO) analysis, are shown in Figure 4B qPCR results further verified that kumatakenin significantly increased enolase (Eno3) expression in colitis mice (Figure 4C).

To further verify whether Eno3 was involved in kumatakenin-induced anti-ferroptosis effects, protein expression of Eno3 in kumatakenin-treated colitis mice was measured. Western blotting and IHC confirmed that Eno3 expression in colitis mice was significantly lower than the vehicle group; this effect was reversed by treatment with kumatakenin (Figures 4D,E).

**FIGURE 5:**
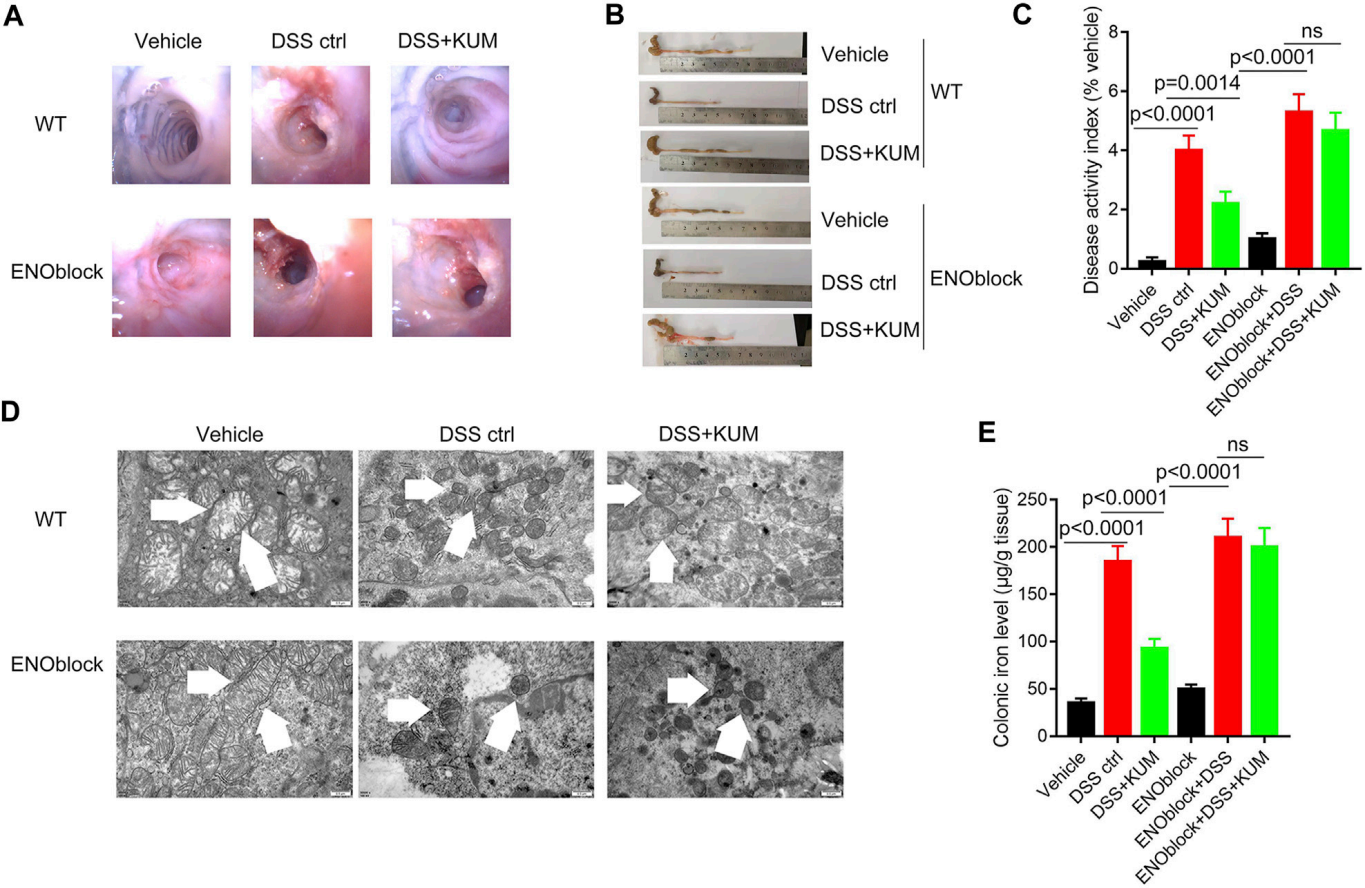
Knockdown of Eno3 significantly reversed kumatakenin-induced regulation of epithelial ferroptosis in colitis mice. Mice were administered the ENO inhibitor ENOblock by intraperitoneal injection 3 days before DSS or kumatakenin treatment, and the effects of kumatakenin on **(A)** vascular patterns, atrophy of intestinal villi, **(B)** colon length, **(C)** disease activity index, **(D)** epithelial mitochondrial morphology (scale bar = 0.5 μm) and **(E)** cellular iron levels in epithelial cell in colitis mice were measured. KUM, kumatakenin.

Furthermore, the Eno3 inhibitor ENOblock significantly reversed kumatakenin-induced anti-colitis effects, including restoration of mucosal bleeding, vascular patterns and loose stools, restoration of intestinal villi, increased colon length, and decreased DAI score (Figures 5A–C). ENOblock treatment also reversed kumatakenin-induced suppression of ferroptosis, characterized by restoration of mitochondrial morphology and reduction of colonic iron levels in colitis mice (Figures 5D, E). These data showed that kumatakenin inhibited ferroptosis in epithelial cells from colitis mice in an Eno-3-dependent manner.

### Kumatakenin reduced iron levels by modulating the Eno3-IRP1 axis

**FIGURE 6:**
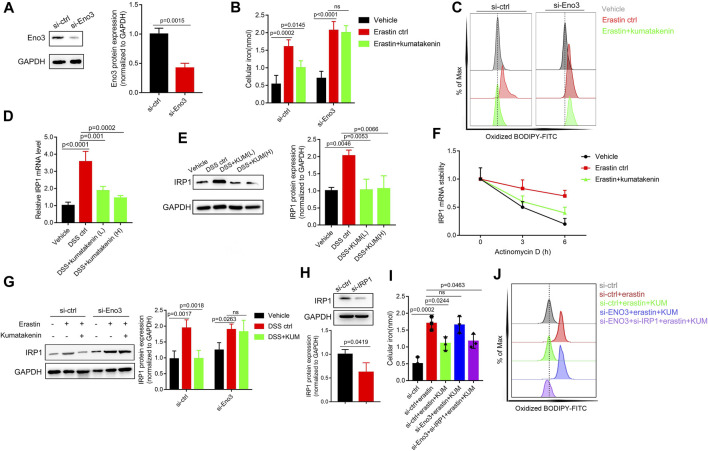
Kumatakenin reduced cellular iron levels by activating Eno-3 and inducing degradation of iron regulatory protein 1 (IRP1) mRNA. **(A)** Examination of Eno3 expression to verify the knockdown efficiency of siEno3. Effects of Eno3 knockdown on kumatakenin-exerted effects on **(B)** cellular iron levels and **(C)** lipid ROS *in vitro*. **(D,E)** Effects of kumatakenin on colonic IRP1 mRNA and protein expression in colitis mice. **(F)** IRP1 mRNA stability was determined in erastin-treated MODE-K cells by incubating with the transcription inhibitor actinomycin D (5 μM). **(G)** Effects of knockdown of Eno3 on IRP1 expression. **(H)** Examination of IRP1 expression to verify the knockdown efficiency of si-IRP1. Effects of Eno3 knockdown and IRP1 on cellular iron levels **(I)** and lipid ROS **(J)**.

Small interfering mediated Eno-3 gene knockdown in MODE-K cells was performed to further measure the mechanism underlying kumatakenin-induced suppression of iron levels and ferroptosis in epithelial cells. Our results showed that kumatakenin reversed the increased iron levels and lipid peroxidation induced by erastin. Knockdown of Eno-3 significantly abolished kumatakenin-exerted suppression of iron content and lipid peroxidation level (Figures 6A–C), consistent with the *in vivo* results.

A previous study showed that upregulation of Eno family members led to inactivation of iron-regulatory protein 1 (IRP1), decreased iron levels, and suppression of ferroptosis (Zhang et al., 2022). ENO, as an RNA-binding protein, binds the IRP1-5′ untranslated region (UTR), promoting degradation of IRP1 mRNA (Zhang et al., 2022). Given that IRP1 serves as an important factor involved in iron homeostasis (Huynh et al., 2019), we further measured the effects of kumatakenin on mRNA stability and protein expression of IRP1 in colitis mice. Our results showed that mRNA and protein expression of IRP1 in colonic tissues was significantly increased in colitis mice (Figures 6D,E) compared with the vehicle group. Kumatakenin treatment inhibited the accumulation of IRP1 in the DSS-treated group. RNA stability assays showed that erastin treatment significantly inhibited IRP1 mRNA degradation in MODE-K cells (Figure 6F), which could be reversed by kumatakenin treatment.

To further confirm whether kumatakenin decreased IRP1 expression by regulation of Eno3, we measured the effects of kumatakenin on IRP1 in the erastin-treated group. Our results showed that IRP1 expression was increased after erastin treatment, and that this effect could be reversed by kumatakenin. Knockdown of Eno3 significantly blocked kumatakenin-exerted regulation of IRP1 (Figure 6G), indicating kumatakenin inhibited IRP1 expression by activating Eno3. Furthermore, knockdown of Eno3 significantly abolished kumatakenin-mediated regulation of iron and lipid reactive oxygen species (ROS) levels in epithelial cells; these effects were reversed by genetic knockdown of IRP1 (Figures 6H, I). Taken together, these data showed that kumatakenin inhibited cellular iron levels and suppressed ferroptosis in epithelial cells by regulating the Eno-3-IRP1 axis.

### Molecular binding between kumatakenin and Eno3

**FIGURE 7:**
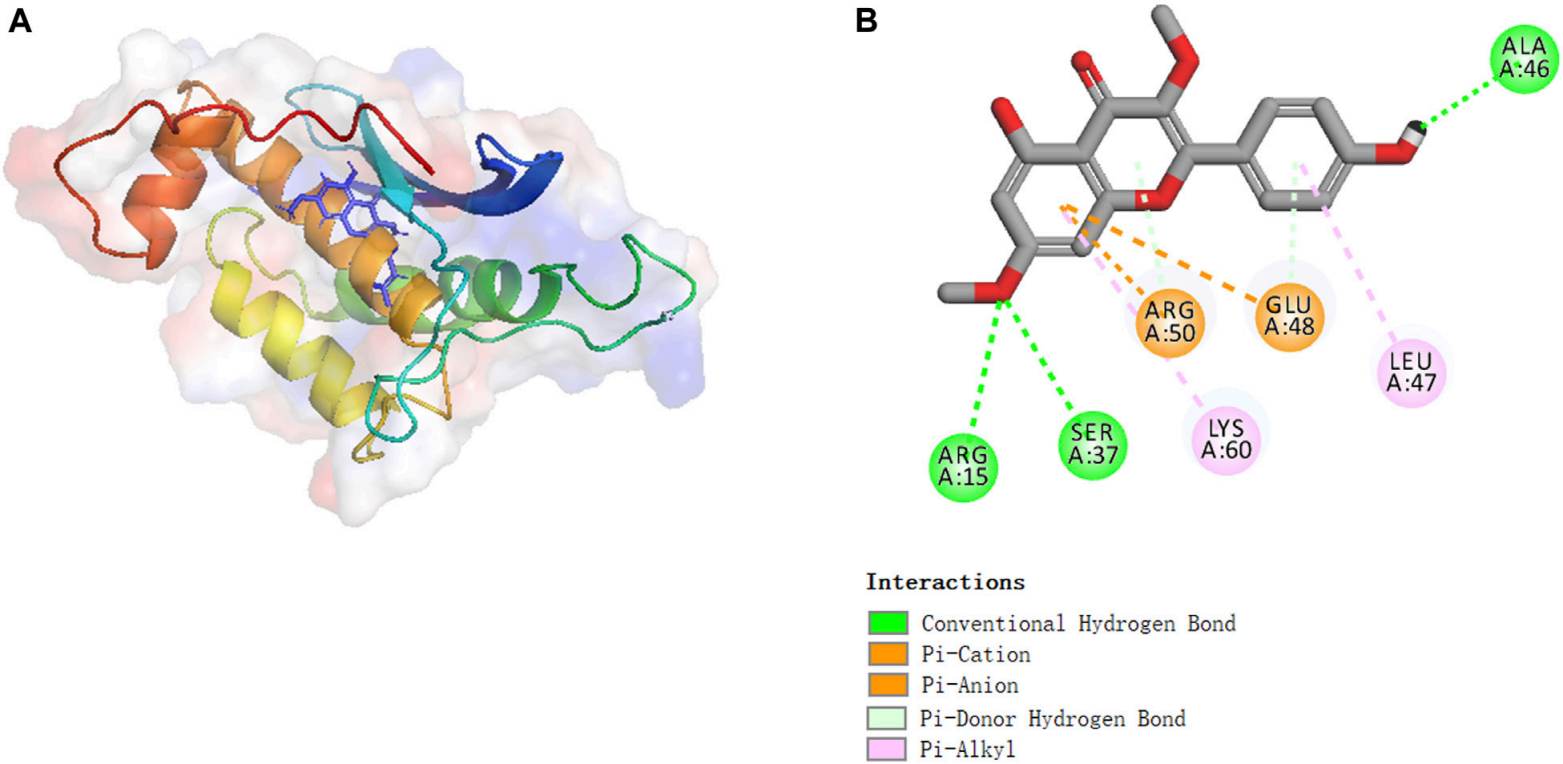
Molecular binding between kumatakenin and Eno3. Molecular docking experiments were performed as shown. **(A)** 3D structures and **(B)** 2D interactions of kumatakenin with Eno3.

Molecular docking experiments were performed to investigate the activation mechanism between kumatakenin and Eno3. Figure 7 shows that kumatakenin can bind Eno3. Kumatakenin formed three hydrogen bonds with the residues in Eno3. Hydroxyl groups at C-1′, C-2″, and C-2″ formed hydrogen bonds with Ala46, Ser37, and Arg15, respectively. Thus, Ala46, Ser37, and Arg15 were identified as contact sites in Eno3 bound to kumatakenin.

## Discussion

In epithelial cells, ferroptosis, a type of programmed cell death characterized by accumulation of lipid peroxidation products, contributes to the development of colitis. Inhibition of ferroptosis proved to be an attractive approach for treatment of IBD. Kumatakenin is a polymethoxylated flavonoid isolated from traditional Chinese medicinal cloves and *Alpinia purpurata* (Villaflores et al., 2010; Woo et al., 2017). Previous work has confirmed that kumatakenin exerts significant anti-inflammatory effects, and induced cell death of cancer cell lines. However, the effects of kumatakenin on DSS-induced colitis and whether kumatakenin influences colitis by inhibiting epithelial ferroptosis remain unclear.

Our work confirmed that kumatakenin treatment significantly alleviated DSS-induced colitis at least largely by inhibiting epithelial ferroptosis in colonic tissues. Kumatakenin treatment upregulated Eno3 expression, resulting in degradation of IRP1 mRNA and downregulation of IRP1 expression. Due to the regulatory role of IRP1 on cellular iron levels, kumatakenin-exerted suppression of IRP1 contributed to homeostasis of cellular iron levels and inhibition of epithelial ferroptosis.

Natural products possess a number of advantages (fewer side effects, less cell toxicity, more economical, and multi-target) in the treatment of diseases compared with clinically used drugs. Thus, natural products are regarded as promising resources for IBD drug discovery. Due to the significant alleviation of colitis mediated by kumatakenin and lack of significant toxicity in normal mice/cell lines, this flavonoid is an attractive candidate drug for IBD therapy.

The exact mechanism underlying kumatakenin-induced activation of Eno3 protein remains elusive. Alteration of mRNA transcription leads to variation in the expression of protein (Hausser et al., 2019). Modification of protein stability also directly influences protein expression (Goldenzweig et al., 2016). Thus, future studies will investigate whether kumatakenin influences Eno3 transcription and/or protein stability.

Overall, kumatakenin alleviated DSS-induced colitis largely by inhibiting epithelial ferroptosis, which was linked to activation of Eno3 and subsequent degradation of IRP1 and homeostasis of cellular iron levels. This work may provide new guidance for the clinical use of kumatakenin to treat colitis.

## Data Availability

The datasets presented in this study can be found in online repositories. The names of the repository/repositories and accession number(s) can be found below: https://www.ncbi.nlm.nih.gov/bioproject/PRJNA913780.

## Supplementary material

SUPPLEMENTARY FIGURE S1
Chemical formula of kumatakenin and toxicity of kumatakenin in normal mice and MODE-K cells. **(A)** Cell toxicity of kumatakenin. Effects of kumatakenin on **(B)** body weight, **(C)** disease activity index, **(D)** pro inflammatory cytokine TNF-α production, **(E)** serum FD-4, **(F)** colonic iron level, and **(G)** Eno3 expression in normal mice.

SUPPLEMENTARY TABLE S1
Source data of molecular binding between kumatakenin and Eno3.
