# Supplementary figures and images for: Kumatakenin inhibited iron-ferroptosis in epithelial cells from colitis mice by regulating the Eno3-IRP1-axis
Source: Front Pharmacol. 2023 Mar 17;14:1127931. doi: 10.3389/fphar.2023.1127931 (PMC10063804; doi:10.3389/fphar.2023.1127931)

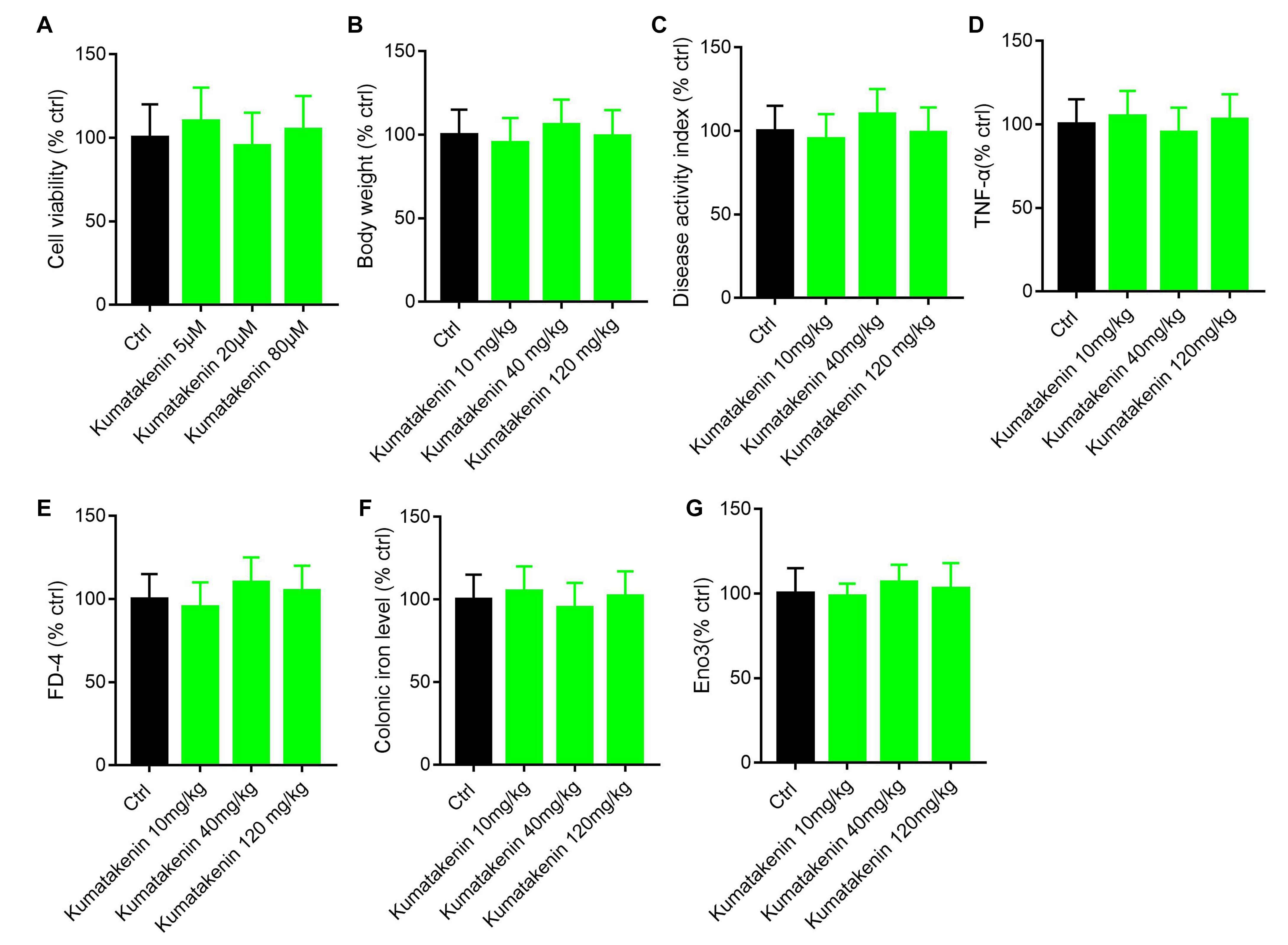

Supplement: Supplementary file 1 [file Image1.TIF]
